# Z-Scheme MIL-53(Fe)/Mn-SrTiO_3_ heterostructure for efficient photocatalytic degradation of o-nitrophenol

**DOI:** 10.1098/rsos.250768

**Published:** 2025-11-12

**Authors:** Mirna Omar, Sarah Omar, Gehan M. El-Subruiti, Nour F. Attia, Abdelazeem Eltaweil

**Affiliations:** ^1^ Department of Chemistry, Faculty of Science, Alexandria University, Alexandria, Egypt; ^2^ Department of Mechanical Engineering, Universiti Teknologi Petronas, Seri Iskandar, Malaysia; ^3^ Department of Gas Analysis and Fire Safety Laboratory, Chemistry Division, National Institute for Standards, Egypt 136 Giza 12211, Egypt; ^4^ Department of Engineering, University of Technology and Applied Sciences, Muscat, Oman

**Keywords:** Z-scheme, SrTiO_3_, degradation, MIL-53(Fe), o-nitrophenol

## Abstract

With growing concerns over water pollution and depletion of freshwater resources, finding sustainable and cost-effective solutions for water treatment is crucial. This study introduces a novel MIL-53(Fe)/Mn-doped SrTiO_3_ (MIL-53(Fe)/Mn-STO) direct Z-scheme photocatalyst, designed to efficiently degrade organic contaminants in water. Specifically, the photocatalytic degradation of o-nitrophenol (ONP) was investigated under an Xe lamp. The MIL-53(Fe)/Mn-STO photocatalyst demonstrated a maximum removal efficiency of 97.75% within 90 min, with a rate constant (*k*) of 0.0374 min^−1^, outperforming the individual MIL-53(Fe) and Mn-STO photocatalysts. A comprehensive analysis of the material’s properties was conducted using Fourier transform Infrared spectroscopy, energy dispersive X-ray spectroscopy, scanning electron microscopy, diffuse reflectance spectroscopy and photoluminescence techniques, and the photocatalytic activity was evaluated under various conditions, including variations in pH, ONP concentration, catalyst dosage and the presence of inorganic anions. The improved photocatalytic activity of the MIL-53(Fe)/Mn-STO system can be ascribed to the synergistic interaction between its two components, which also contributed to its excellent recyclability over five cycles. These findings demonstrate that MIL-53(Fe)/Mn-STO is a highly effective and sustainable photocatalyst with a strong potential for wastewater treatment applications.

## 1. Introduction

Water pollution poses a critical threat to global ecosystems and human health, primarily due to the discharge of hazardous industrial effluents into natural water bodies [1]. Among these pollutants, o-nitrophenol (ONP) is particularly concerning due to its widespread presence in wastewater from industries such as dye production, pharmaceuticals and agrochemicals [2]. ONP is chemically stable, highly persistent in aquatic environments and exhibits both acute and chronic toxicity to humans and ecosystems [3]. It is harmful if swallowed, inhaled or absorbed through the skin. Exposure may cause irritation of the eyes, skin and respiratory tract, as well as methaemoglobinaemia, an oxygen-transport disorder that can lead to respiratory distress, convulsions and neurological complications [2]. Direct contact can cause ocular damage, including corneal opacity and cataracts [4]. Environmentally, nitrophenols are persistent and highly toxic to aquatic life, disrupting the ecosystem balance and threatening biodiversity [5]. Therefore, it is crucial to develop effective and sustainable methods for removing ONP from water sources to mitigate its harmful effects and protect aquatic ecosystems. Among the various techniques, advanced oxidation processes, including ozonation and the photo-Fenton method, have shown promise for ONP degradation [6]. However, photocatalysis is emerging as a more efficient and sustainable technology, offering complete mineralization of pollutants using light energy [7,8]. The development of highly effective photocatalysts for environmental clean-up, particularly for the degradation of organic contaminants, has garnered significant interest [7]. Semiconductor-based photocatalysis is particularly promising for water purification because it can degrade a wide range of organic pollutants under light irradiation [9]. Among various photocatalysts, metal–organic frameworks (MOFs) and perovskite materials have gained attention as potential candidates owing to their distinctive structural features and enhanced photocatalytic activities [10,11]. MOFs, such as MIL-53(Fe), offer large surface areas, tunable porosity and adjustable chemical functionalities, which are advantageous for photocatalytic applications [12]. In contrast, perovskite materials such as strontium titanate (SrTiO_3_), particularly when doped with transition metals such as manganese (Mn), exhibit excellent light absorption and charge separation characteristics, rendering them effective in photocatalytic applications [13]. However, the photocatalytic efficiency of single MOF or perovskite materials remains relatively low, mainly because of their limited absorption spectrum and the rapid recombination of photoexcited charge carriers [14]. To overcome these issues, researchers have focused on modifying these materials through metal doping, surface modification or constructing heterojunctions to improve their photocatalytic efficiency. Among various strategies, constructing heterojunctions between MOFs and perovskites has proven to be an effective approach for boosting photocatalytic activity [15]. The synergistic integration of these materials into a Z-scheme heterojunction allows for efficient charge transfer and separation, reducing recombination rates and improving photocatalytic performance under visible light [16]. This study focuses on the development and analysis of a newly designed composite photocatalyst composed of MIL-53(Fe) and Mn-doped SrTiO_3_ (Mn-STO). MIL-53(Fe)/Mn-doped SrTiO_3_ (MIL-53(Fe)/Mn-STO) composite was synthesized via a solvothermal approach, and its photocatalytic performance was evaluated for the degradation of ONP. The combination of MIL-53(Fe) and Mn-STO was engineered to exploit the strengths of both materials. MIL-53(Fe) has a high surface area and adjustable porosity, providing numerous active sites for photocatalytic reactions [17]. In contrast, Mn-STO improves light absorption and charge carrier mobility owing to its optimal band alignment and doping effects, resulting in improved photocatalytic activity under visible-light exposure [13].

This study aimed to develop and investigate a novel Z-scheme MIL-53(Fe)/Mn-SrTiO_3_ photocatalyst for the efficient degradation of ONP in water. This study focuses on enhancing photocatalytic performance through synergistic charge transfer mechanisms, optimizing reaction conditions and evaluating the structural, optical and chemical properties of the catalyst using Fourier transform Infrared spectroscopy (FT-IR), energy dispersive X-ray spectroscopy (EDX), scanning electron microscopy (SEM), diffuse reflectance spectroscopy (DRS) and photoluminescence (PL) analyses. Additionally, the stability, recyclability and degradation efficiency of the photocatalyst under various environmental conditions were assessed.

## 2. Experimental section

### 2.1. Materials

Titania TiO_2_ (P25) powder was sourced from Sigma-Aldrich Co. Sr(OH)_2_.8H_2_O, KOH, MnCl_₂_.4H_2_O and ethanol were acquired from EL NASR Pharmaceutical Company, Egypt. Terephthalic acid (TPA), FeCl_3_·6H_2_O and N,N-dimethylformamide (DMF) were provided by Aladdin Reagent Co., Ltd (China).

### 2.2. Synthesis of photocatalysts

A two-step process was employed to synthesize the MIL-53(Fe)/Mn-STO nanocomposite photocatalyst designed for the degradation of organic contaminants under Xe lamp irradiation. Initially, a hydrothermal reaction was performed, followed by a solvothermal reaction.

### 2.3. Preparation of Mn-doped SrTiO_3_

Mn-STO was synthesized using a hydrothermal approach. In a standard procedure, 0.14 g of MnCl_2_·4H_2_O was mixed with 10 ml of deionized water and slowly introduced with stirring into a solution containing 2 g of SrCl_2_.6H_2_O and 2 g of TiO_2_ in 30 ml of purified water. Subsequently, 2M KOH solution was introduced to the blend to adjust and stabilize the pH to 13, after which the solution was agitated vigorously for 30 min. The resulting mixture was transferred to a 50 ml Teflon-lined stainless-steel container, where the hydrothermal process was conducted at 150℃ over a span of 3 days. After natural cooling to room temperature, the resulting deep-green solid was collected via centrifugation. The material was then washed several times with purified water and ethanol, and subsequently dried under vacuum at 60℃ for 12 h, according to previous reports [18].

### 2.4. Synthesis of MIL-53(Fe)/Mn-doped SrTiO_3_

**Scheme 1.**
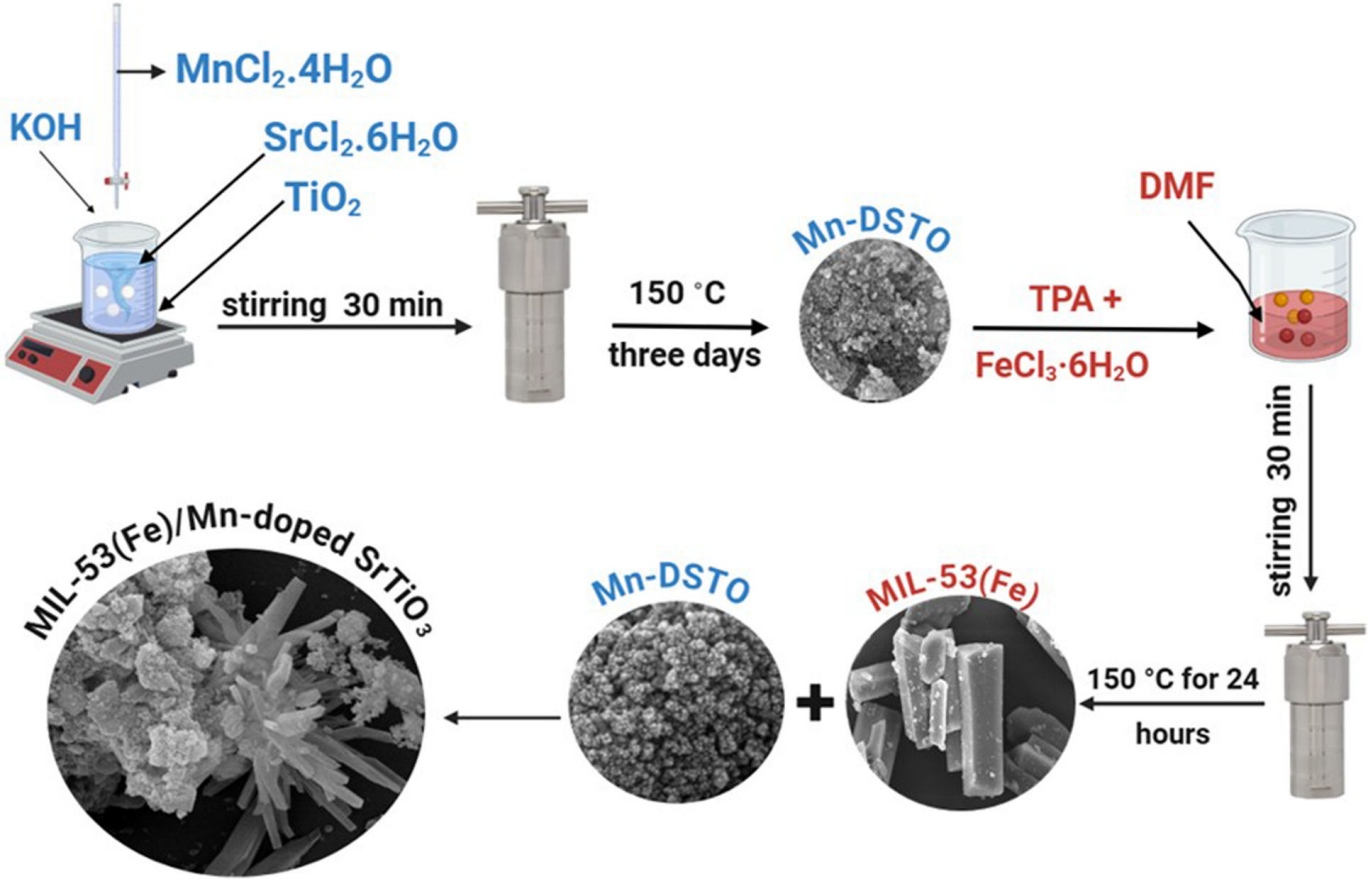
Schematic of MIL-53(Fe)/Mn-STO photocatalyst synthesis.

The photocatalyst was prepared using a solvothermal method. In a typical procedure, 1.66 g of TPA, 2.70 g of FeCl_3_·6H_2_O and 2.70 g of Mn-STO were mixed with 56 ml of DMF as the solvent. The mixture was stirred at room temperature for 30 min to ensure homogeneity. The solution was then transferred to a 100 ml stainless-steel autoclave and heated at 150℃ for 24 h. After cooling to room temperature, the product was separated by centrifugation at 8000 r.p.m. for 4 min, washed three times with DMF and absolute ethanol and finally dried under reduced pressure at 60℃ for 12 h, as shown in Scheme 1. Pristine MIL-53(Fe) was synthesized using the same procedure according to previous reports [19], omitting the addition of Mn-STO. The obtained composite is hereafter referred to as MIL-53(Fe)/Mn-STO.

### 2.5. Characterization methods

The characterization techniques used are detailed in the electronic supplementary material, text S1.

### 2.6. Photocatalytic breakdown experiments

Information regarding the photodegradation process can be found in the electronic supplementary material, text S2.

## 3. Results and discussion

### 3.1. Structural and morphological analysis of the developed photocatalysts

**Figure 1.**
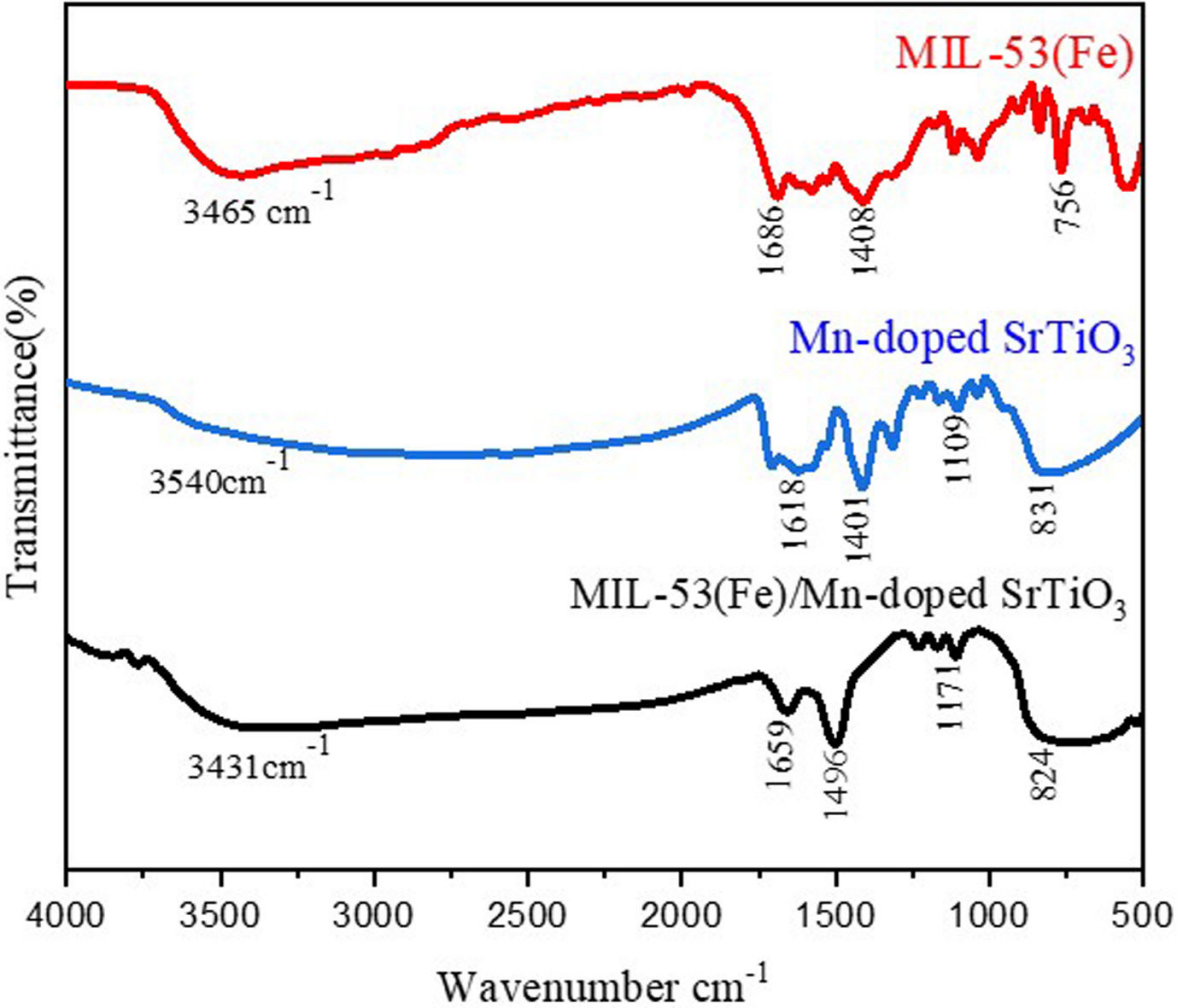
FT-IR spectra of MIL-53(Fe), Mn-doped SrTiO_3_ and the composite MIL-53(Fe)/Mn-doped SrTiO_3_ nanocatalysts.

**Figure 2.**
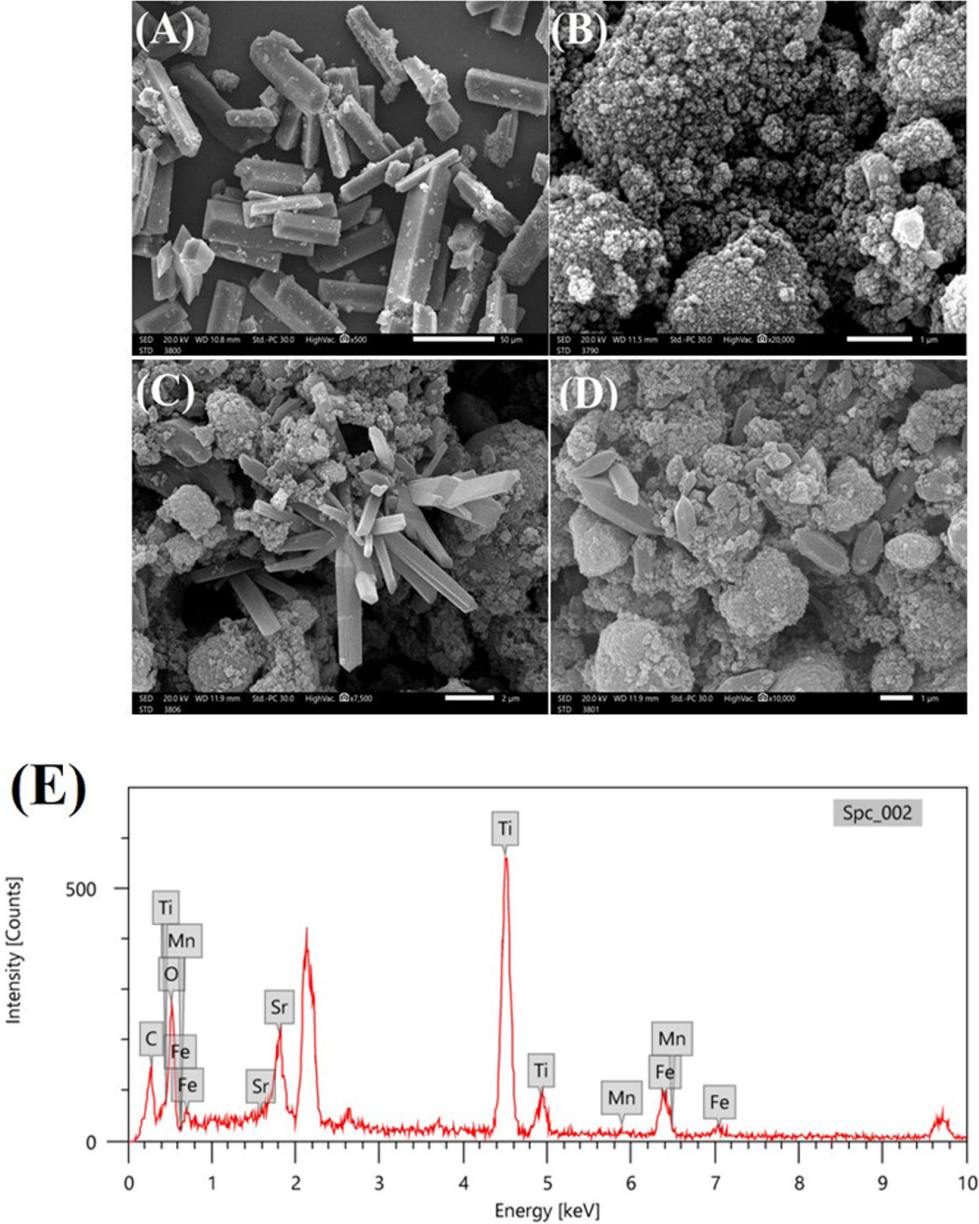
SEM image (A) depicts well-defined rod-like structures of MIL-53(Fe), while (B) shows the agglomerated granular structure of Mn-STO. Images (C) and (D) showcase the composite material, highlighting the integration of MOF and perovskite structures with mixed morphologies, indicating successful synthesis and uniform distribution of the composite components. (E) The EDX spectrum of MIL-53(Fe)/Mn-STO photocatalyst.

Figure 1 shows the FT-IR spectra of MIL-53(Fe), Mn-STO and MIL-53(Fe)/Mn-STO composite. The broad band observed at 3465–3431 cm^−1^ corresponds to the O–H stretching vibration, which is attributed to moisture adsorbed on the surface of all the synthesized samples [20]. In both Mn-STO and the MIL-53(Fe)/Mn-STO composite, the band at 1659–1618 cm^−1^ is assigned to the bending (scissoring) vibration of H_2_O molecules [20]. For MIL-53(Fe), the bands observed at 1686 and 1408 cm^−1^ can be assigned to the asymmetric and symmetric stretching vibrations of carboxylate (–COO^−^) groups from the terephthalate linkers in the MOF framework [21,22]. Additionally, a distinct peak at 756 cm^−1^ arises from the out-of-plane C–H bending of the aromatic ring in the terephthalate linker [23]. In the spectrum of Mn-STO, the band at 1401 cm^−1^ is more reliably assigned to surface carbonate species, particularly the symmetric CO_3_^2−^ stretch and C–O bending, commonly formed due to CO_2_ adsorption on titanate surfaces [24]. The band at 1109 cm^−1^ can be attributed to Ti–OH stretching, and the peak near 831 cm^−1^ most likely corresponds to Ti–O–Ti lattice vibrations and possibly out-of-plane carbonate modes [25,26]. The MIL-53(Fe)/Mn-STO composite spectrum exhibits characteristic peaks from both parent components, but with slight shifts and intensity variations. Notably, the carboxylate stretching bands appear at 1659 cm^−1^ (asymmetric) and 1496 cm^−1^ (symmetric), confirming the preservation of the MIL-53(Fe) framework. The peak at 824 cm^−1^ corresponds to Ti–O bending vibrations in the perovskite lattice. The observed shifts in the band positions compared with those of materials indicate strong interfacial interactions between MIL-53(Fe) and Mn-STO, which support the successful formation of the composite heterostructure. The morphological characteristics of MIL-53(Fe), Mn-STO and MIL-53(Fe)/Mn-STO composite were examined using SEM. The SEM image in figure 2A demonstrates that the MIL-53(Fe) MOF defines rod-like structures with relatively smooth surfaces. On the other hand, the SEM image of the Mn-STO perovskite in figure 2B displays a different morphology, characterized by highly agglomerated nanoparticles forming irregular, granular clusters. The distinct morphology of the perovskite particles suggests a significant surface area, potentially enhancing photocatalytic activity by providing more active sites. Based on the SEM image, the composite material exhibited a well-defined morphology characterized by rod-like structures and granular formations. The SEM images in figure 2C,D reveal that the MIL-53(Fe)/Mn-STO composite maintains a robust integration of both the MOF and perovskite phases. The prominent rod-like crystals are indicative of the MIL-53(Fe) framework, while the surrounding granular material suggests the presence of the Mn-STO component. This heterogeneous morphology probably contributes to an enhanced surface area, which offers more reactive centres for photocatalytic reactions. These structural characteristics collectively suggest that the composite can potentially exhibit improved photocatalytic performance, particularly in processes such as the breakdown of organic contaminants. The successful synthesis and integration of MIL-53(Fe) and Mn-STO within the composite were further evidenced by the uniform distribution and interaction of the different phases, as observed in the SEM analysis. The results from the SEM examination aligned well with the EDX data (figure 2E), presenting the elemental make-up of the MIL-53(Fe)/Mn-STO photocatalyst. EDX analysis confirmed the presence and spatial arrangement of all components in the MIL-53(Fe)/Mn-STO material. Based on the detected peaks, the elemental compositions by weight percentages were ranked as follows: O > Ti > C > Fe > Sr > Mn. Electronic supplementary material, figure S1, provides additional confirmation of the effective preparation of MIL-53(Fe)/Mn-STO photocatalyst.

### 3.2. Optical characteristics of the synthesized nanocomposite photocatalyst

**Figure 3.**
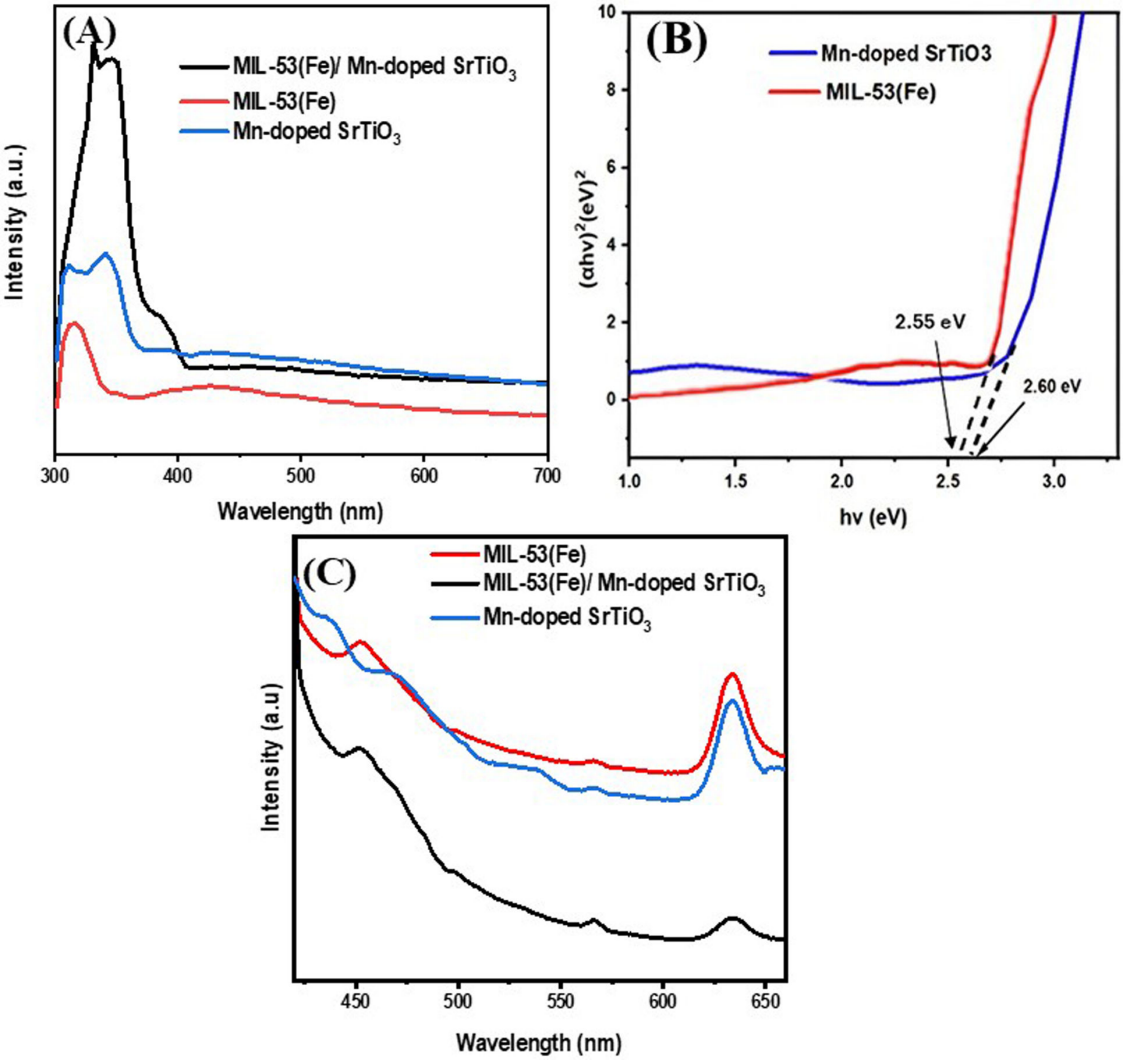
(A) UV-visible DRS of MIL-53(Fe)/Mn-doped SrTiO_3_, MIL-53(Fe) and Mn-doped SrTiO_3_. (B) Tauc plot obtained from Kubelka–Munk-transformed DRS data for MIL-53(Fe) and Mn-STO, showing extrapolated optical bandgap energies. (C) PL spectra of MIL-53(Fe)/Mn-STO, MIL-53(Fe) and Mn-STO.

UV-visible DRS was employed to investigate the optical properties of MIL-53(Fe)/Mn-STO nanocomposite. As illustrated in figure 3A, MIL-53(Fe) and Mn-STO exhibited absorption edges at approximately 319 and 326 nm, respectively [27]. In contrast, the MIL-53(Fe)/Mn-STO composite displayed an extended absorption edge at 350 nm, indicating enhanced visible-light absorption owing to the synergistic combination of MIL-53(Fe) and Mn-STO. Because the measurements were collected as diffuse reflectance, the reflectance data (*R*) were converted to the Kubelka–Munk function


(3.1) $$F(R)=\frac{\left(1-R\right)2}{2R}.$$

This is proportional to the absorption coefficient of the samples. The Tauc relation was then applied using *F*(*R*) in place of α


(3.2) $$(F(R)(h\upsilon ){)}^{n}=A(h\upsilon -E_{g}),$$

where *hυ* is the photon energy, *A* is a constant, *E*_*g*_ is the optical bandgap and *n* = 1/2 corresponds to an indirect allowed electronic transition. This choice of *n* is consistent with the literature for similar materials [28,29] and is supported by the quality of the linear fits in the Tauc plots (figure 3B). The optical bandgap *E*_*g*_ was obtained by the extrapolation of the linear portion of the Tauc plot to the photon energy axis. The calculated bandgaps for MIL-53(Fe) and Mn-STO were 2.55 and 2.60 eV, respectively, which are consistent with the reported values [17,30,31]. Determining the optical bandgap is essential for understanding the photocatalytic performance, as it defines the threshold photon energy required to excite electrons from the valence band (VB) to the conduction band (CB). A reduced *E*_*g*_ in the MIL-53(Fe)/Mn-STO composite compared with the pristine components enables more effective harvesting of visible light, which enhances electron–hole pair generation and improves photocatalytic degradation efficiency. The PL spectra of Mn-STO, MIL-53(Fe) and MIL-53(Fe)/Mn-STO were examined to assess the rate at which photogenerated electron–hole pairs recombine. As depicted in the PL spectra (figure 3C), bare MIL-53(Fe) and Mn-STO both demonstrated high bandgap emission intensities, indicative of elevated recombination rates for the photogenerated charge carriers. Notably, the MIL-53(Fe)/Mn-STO composite exhibited a significant reduction in emission intensity, suggesting enhanced electron–hole separation, particularly due to the influence of the Mn-doped SrTiO_3_ component [32,33]. This outcome implies that the composite material could capitalize on the benefits of both MIL-53(Fe) and Mn-STO, potentially resulting in enhanced photocatalytic performance through a synergistic effect that promotes charge separation and minimizes recombination rate. These results align with previously reported trends that correlate lower PL intensities with improved photocatalytic activities [20,34].

### 3.3. Photocatalysis investigation

**Figure 4.**
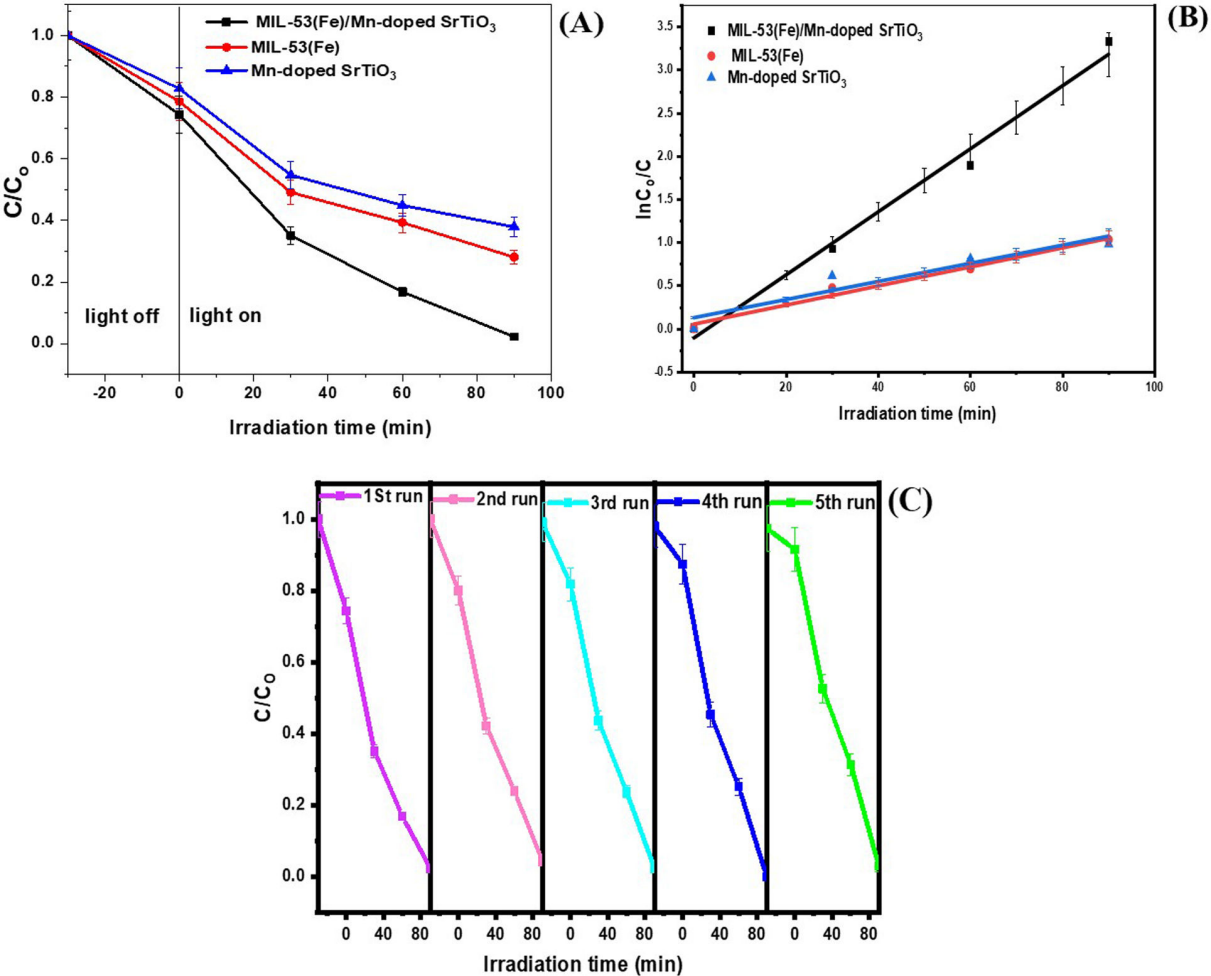
(A) Photocatalytic degradation performance of MIL-53(Fe), Mn-doped SrTiO_3_ and their composite, MIL-53(Fe)/Mn-doped SrTiO_3_. (B) Pseudo-first-order kinetics of ONP photodegradation by MIL-53(Fe), Mn-doped SrTiO_3_ and their composite, MIL-53(Fe)/Mn-doped SrTiO_3_. (C) Recyclability tests of the MIL-53(Fe)/Mn-STO composite for the photocatalytic degradation of pollutants. Error bars represent the s.d. of three independent experiments.

The photocatalytic activity of MIL-53(Fe), Mn-STO and their composite MIL-53(Fe)/Mn-STO was assessed by degrading ONP under an Xe lamp, using a catalyst at a concentration of 0.01 g per 30 ml, with 50 ppm ONP concentration and a pH of 7, and maintained at a temperature of 25°C. The experiment was repeated three times to confirm the reliability of the results. As shown in figure 4A, the MIL-53(Fe)/Mn-STO composite exhibited the highest photocatalytic efficiency, achieving 97.75% degradation of ONP in 90 min. This performance was significantly better than that of MIL-53(Fe) (71.94%) and Mn-STO (62.13%). The improved performance is due to the efficient separation of photogenerated charges and strong visible-light absorption, which is a result of the creation of a direct Z-type heterojunction. Further examination of the kinetics of ONP photodegradation using the MIL-53(Fe)/Mn-STO composite showed a linear relationship in the ln(C_o_/C) versus time plot (figure 4B), indicating that the degradation process adhered to pseudo-first-order reaction kinetics, with correlation coefficients (*R*^²^) above 0.9 for all synthesized samples. The reaction rate constant (*k*) for the MIL-53(Fe)/Mn-STO composite was found to be 0.0374 min^−1^, demonstrating an increase in photocatalytic activity by approximately 3.4 times compared with MIL-53(Fe) (*k* = 0.011 min^−1^) and 4.4 times compared with Mn-STO (*k* = 0.0085 min^−1^). The photostability and recyclability of the MIL-53(Fe)/Mn-STO composite were tested under constant parameters (ONP concentration = 50 mg l^−1^, catalyst dosage = 0.02 g, 25°C). After each cycle, the catalyst was recovered by centrifugation, washed and dried overnight at 70℃. The ONP degradation efficiency of the composite gradually decreased over five cycles, achieving 97.75% in the first run, 95.79% in the second, 88.77% in the third, 81.76% in the fourth and 74.75% in the fifth. These findings, illustrated in figure 4C, indicate that the MIL-53(Fe)/Mn-STO photocatalyst has significant potential for real applications.

### 3.4. Performance assessment

The photocatalytic activity of MIL-53(Fe)/Mn-STO was evaluated to determine optimal photocatalytic degradation performance. All experiments were performed in triplicate to ensure reproducibility, with error bars indicating the s.d.

#### 3.4.1. pH influence

**Figure 5.**
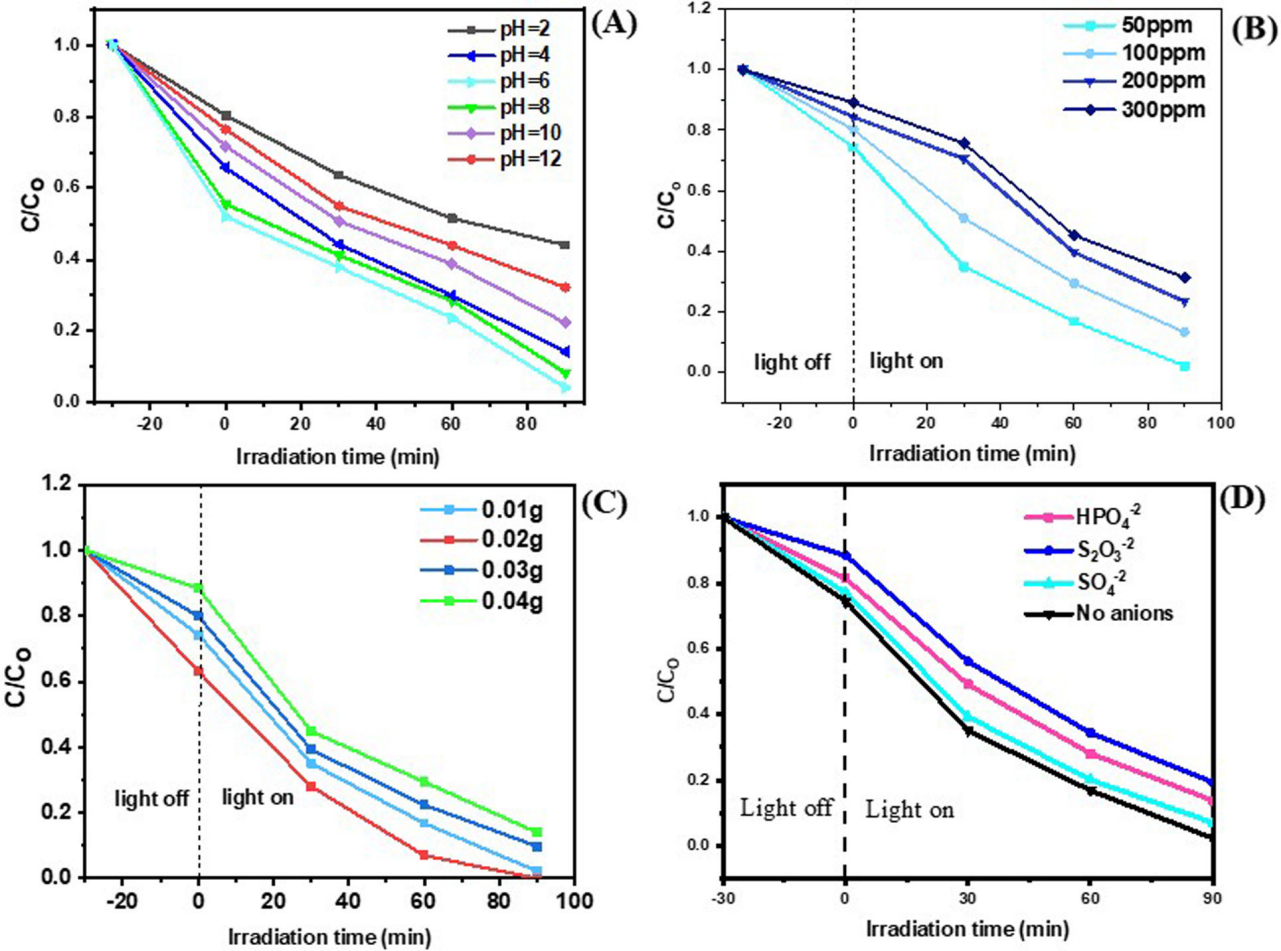
Reduction in ONP concentration (C/Co) using the photocatalyst under varying conditions: (A) different pH levels; (B) various initial concentrations; (C) different photocatalyst dosages; (D) impact of inorganic anions on the MIL-53(Fe)/Mn-STO system for degrading ONP.

The pH of the solution plays an essential role in the photocatalytic degradation mechanism, influencing both the surface charge of the photocatalyst and the ionization state of ONP [35]. In this study, we investigated the influence of pH values from 2 to 12 by modifying the pH values employing 0.01 mol l^−1^ solutions of NaOH and HCl solutions, while keeping all other experimental parameters constant (ONP concentration = 50 mg l^−1^, catalyst dosage = 0.02 g, no added anions). MIL-53(Fe)/Mn-STO point of zero charge (pHpzc) was determined to be pH 7.5. Therefore, at pH levels exceeding this threshold, the surface of the photocatalyst becomes negatively charged, whereas at pH levels below 7.5, it remains positively charged. The chemical structure of ONP changes significantly with pH because of the protonation–deprotonation of its phenolic ^−^OH group (pKa ≈ 7.2). At acidic pH, ONP is predominantly in its neutral molecular form, in which the ^−^OH group is protonated, leading to lower solubility and weaker electrostatic interactions with the positively charged catalyst surface [36]. At near-neutral pH, partial deprotonation occurs, increasing the electron density on the aromatic ring and facilitating oxidative attack by reactive species [37]. At alkaline pH, ONP exists mainly as a phenolate anion, which is more soluble but experiences electrostatic repulsion from the negatively charged catalyst surface when pH > pHpzc. At very high pH (≥ 12), strong repulsion between the phenolate anion and the catalyst surface significantly reduces adsorption efficiency and thus photocatalytic activity [38]. As shown in figure 5A, an improvement in degradation efficiency was noted as the pH increased from 2 to 6, with the highest degradation of 97.75% observed at pH = 7. This can be attributed to the minimal repulsion and enhanced interaction between the catalyst and pollutant. As the pH further increased to 10, the photocatalytic efficiency remained high but slightly decreased due to the increased negative charge on ONP and the MIL-53(Fe)/Mn-STO surface, leading to moderate electrostatic repulsion. At pH 12, a further decrease in the degradation efficiency was observed, owing to the strong repulsion between the doubly negatively charged species and the negatively charged surface of the photocatalyst [39]. These results indicate that MIL-53(Fe)/Mn-STO effectively degrades ONP, particularly under neutral and slightly alkaline conditions, with the highest efficiency recorded at neutral pH.

#### 3.4.2. Impact of initial concentration

In this study, we examined the impact of varying the initial concentration of ONP within the range of 50–300 ppm, while keeping all other parameters constant (pH = 6, catalyst dosage = 0.02 g, no added anions). As illustrated in figure 5B, the efficiency of photocatalytic degradation declined from 97.75% to 86.59%, 76.50% and 68.51% with a gradual increase in ONP concentration from 50 to 100, 200 and 300 mg l^−1^, respectively. This decline can be attributed mainly to the saturation of available active sites on the MIL-53(Fe)/Mn-STO photocatalyst surface at higher ONP concentrations. Once most of the surface-active sites are occupied, additional ONP molecules cannot be adsorbed effectively, which limits their subsequent photodegradation. Additionally, at higher pollutant concentrations, the thicker ONP layer adsorbed on the catalyst surface can reduce light penetration to the active sites, slightly limiting photon absorption and the generation of reactive species.

#### 3.4.3. Catalyst dosage effect

As depicted in figure 5C, the photocatalytic breakdown of ONP increased from 97.75 to 100% as the dosage of photocatalyst increased from 0.01 to 0.02 g, respectively, while keeping all other experimental parameters constant (ONP concentration = 50 mg l^−1^, pH = 6, no added anions). This effect can be ascribed to an increase in the number of active sites, which enhances both light absorption and reaction opportunities, alongside an increase in the production of free electrons within the CB. However, higher than 0.02 g, the photocatalytic degradation declined due to clustering of particles, which reduced the available surface area and blocked incident light, thereby decreasing efficiency.

#### 3.4.4. Effect of inorganic anions

To further assess the effectiveness of MIL-53(Fe)/Mn-STO, we tested ONP degradation in the presence of various inorganic salts, while keeping all other experimental parameters constant (ONP concentration = 50 mg l^−1^, pH = 6, catalyst dosage = 0.02 g). As illustrated in figure 5D, the inclusion of HPO_4_^2−^, S_2_O_3_^2−^ and SO_4_^2−^ slightly diminished the degradation efficiency of ONP. Nevertheless, the photocatalytic degradation efficiency remained above 80%. The presence of HPO_4_^2−^ may interact with the photocatalyst surface, potentially affecting the generation and recombination of positive holes and superoxide radicals [40]. Thiosulfate ions (S_2_O_3_^2−^) quench both positive holes (^•^h^+^) and superoxide radicals (^•^O_2_^−^) [41]. Additionally, sulphate ions (SO_4_^2−^) can alter the ionic strength of the solution, which may indirectly affect the stability and availability of these reactive species by modifying the overall reaction environment [42].

#### 3.4.5. The photocatalytic degradation mechanism

**Figure 6.**
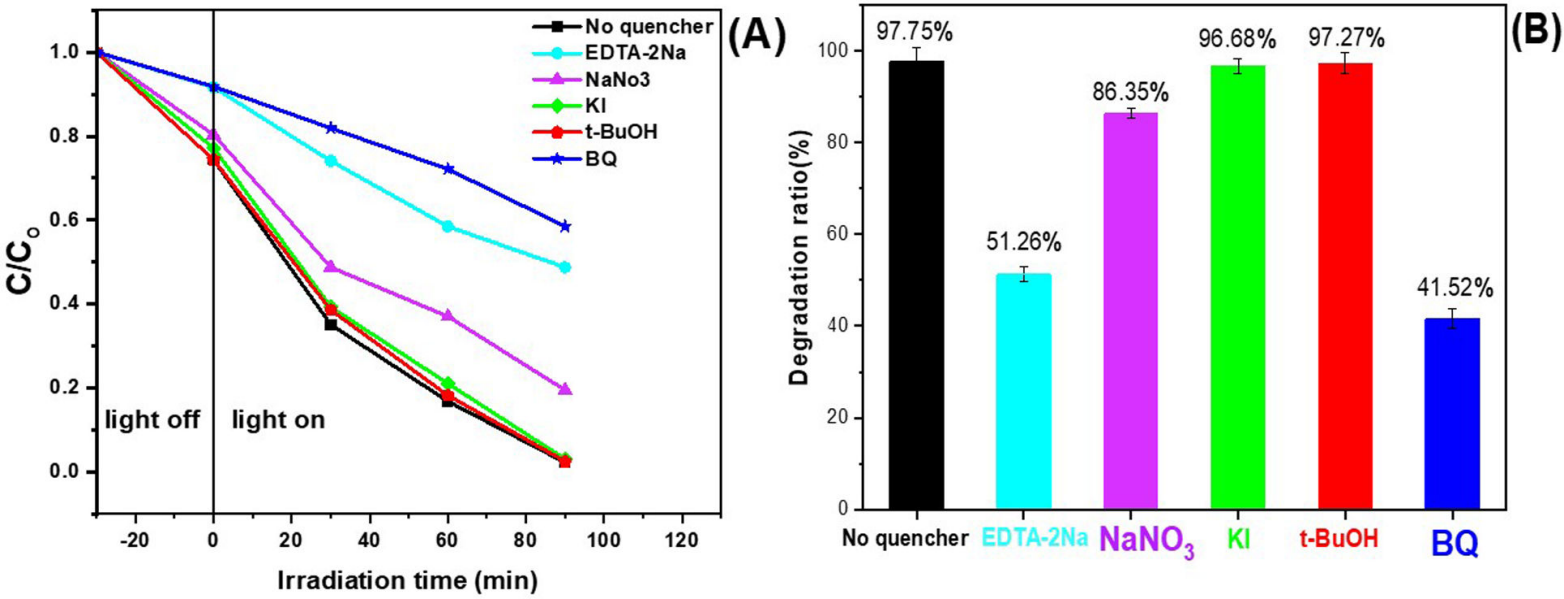
Impact of various scavengers on ONP photodegradation using MIL-53(Fe)/Mn-STO photocatalyst: (A) degradation profile and (B) degradation efficiency.


The underlying mechanism of the photocatalytic reaction was elucidated through radical-trapping experiments, which identified the key reactive species involved in using the MIL-53(Fe)/Mn-STO catalysFigure 6A Under consistent conditions, 3 mM of five different quenchers were separately added to trap specific radicals: KI was used as a scavenger for both free and adsorbed ^•^OH radicals, t-BuOH for free ^•^OH radicals, NaNO_3_ to investigate surface-bound ^•^OH radicals and holes (h^+^), ethylenediaminetetraacetic acid disodium salt (EDTA-2Na) was employed as a hole scavenger and benzoquinone (BQ) was used as an inhibitor to trap superoxide anions (^•^O_2_^−^). As illustrated in figure 6B, the elimination efficiency of ONP was significantly reduced after the addition of EDTA-2Na and BQ. Without any quenchers, the degradation efficiency was 97.75%; however, it declined sharply to 41.52% when BQ was used and to 51.26% in the presence of EDTA-2Na. In contrast, the efficiency decreased slightly to 96.91, 8.5 and 97.4% upon the addition of KI, NaNO_3_ and t-BuOH, respectively. This behaviour is because the VB levels of both MIL-53(Fe) and Mn-STO are not sufficiently positive to achieve the potential required to oxidize H_₂_O into OH radicals. Therefore, the radical scavenging results indicate that ^•^O_2_^−^ and h^+^ are the primary reactive species responsible for ONP degradation. To further elucidate the photocatalytic mechanism of MIL-53(Fe)/Mn-STO, the VB and CB edge potentials of MIL-53(Fe) and Mn-STO were calculated using the following equations:


(3.3) $$E_{CB}=X-E^{e}-\frac{1}{2}E_{g}$$

(3.4) $$E_{VB}=E_{CB}+E_{g}.$$

**Scheme 2.**
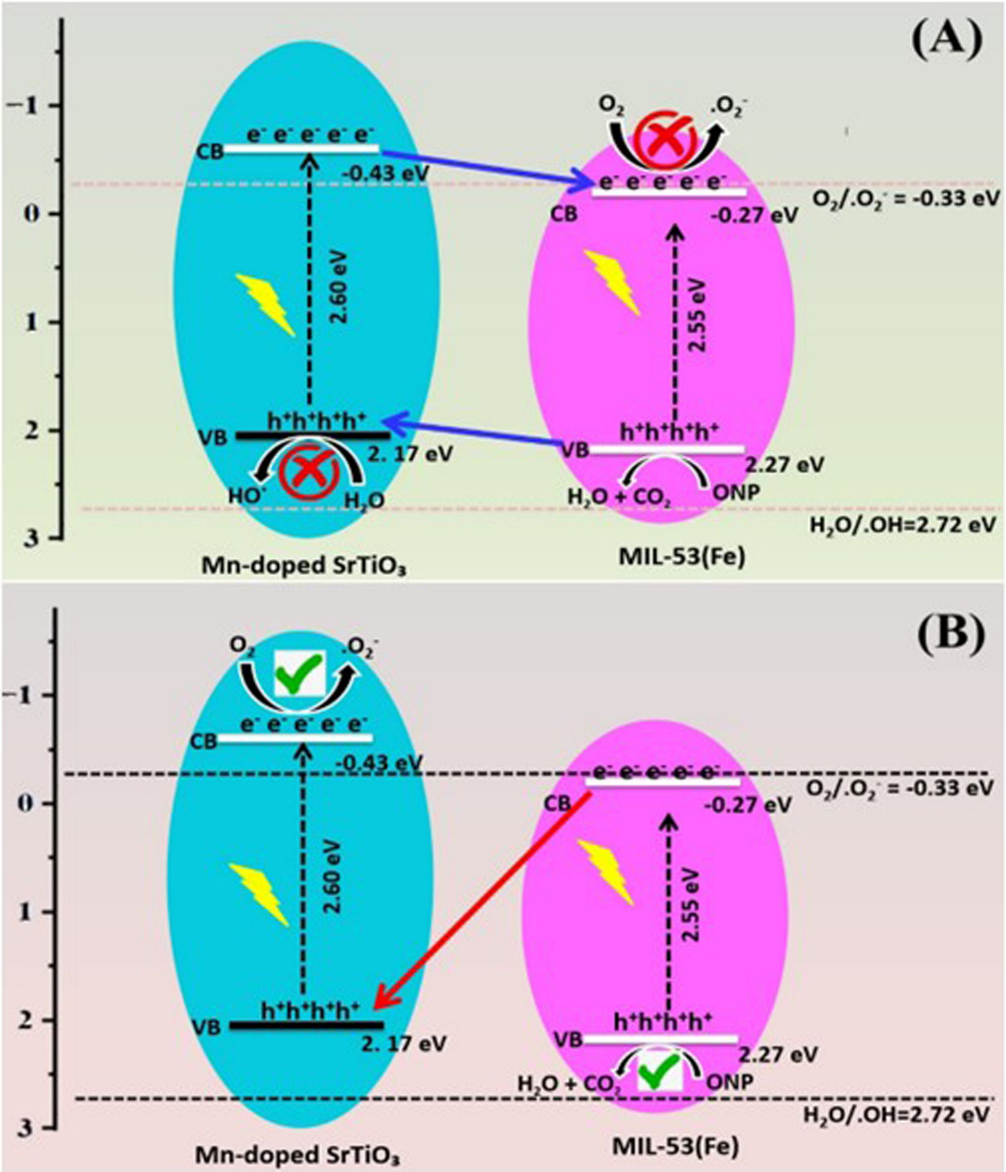
Photodegradation process and possible charge migration mechanisms (A and B).

Here, *X* denotes the geometric mean of the semiconductor’s absolute electronegativity, *E*_*g*_ refers to the bandgap and *E* indicates the energy of free electrons relative to the normal hydrogen electrode (NHE) scale (4.5 eV versus NHE) [43]. The *X* values for MIL-53(Fe) and Mn-STO are approximately 5.5 [31] and 5.37 eV [44], respectively. Based on the bandgap values calculated using UV-visible spectra, MIL-53(Fe) has a bandgap of 2.55 eV, whereas Mn-STO has a bandgap energy of 2.60 eV. Accordingly, the VB and CB positions for MIL-53(Fe) were estimated to be +2.27 eV (VB) and −0.27 eV (CB), whereas those for Mn-STO were +2.17 eV (VB) and −0.43 eV (CB). Based on these results, the MIL-53(Fe)/Mn-STO composite could follow two possible photodegradation mechanisms under Xe lamp illumination, as shown in scheme 2. In the type II heterojunction model (scheme 2A), both MIL-53(Fe) and Mn-STO were excited, generating electrons and holes. Since the CB of MIL-53(Fe) (−0.27 eV) is less negative than that of Mn-STO (−0.43 eV), and the VB of Mn-STO (2.17 eV) is slightly lower than that of MIL-53(Fe) (2.27 eV), the electrons in Mn-STO would migrate to MIL-53(Fe), and the holes in MIL-53(Fe) would migrate to Mn-STO. While this configuration could improve charge separation, the CB of MIL-53(Fe) is not sufficiently negative to reduce O_2_ to ^•^O_2_^−^ (−0.33 eV versus NHE [45]), which conflicts with the radical-trapping results. Instead, a direct Z-scheme mechanism (scheme 2B) is more consistent with the experimental data. In this pathway, photoexcited electrons in the CB of MIL-53(Fe) recombine with holes in the VB of Mn-STO [46,47], retaining electrons in the CB of Mn-STO and holes in the VB of MIL-53(Fe). The retained electrons (−0.43 eV) were sufficiently negative to reduce O_2_ to ^•^O_2_^−^, while the retained holes (2.27 eV) directly oxidized ONP. The inability of MIL-53(Fe) VB holes to oxidize water to ^•^OH radicals (+2.72 eV versus NHE [48]) explains why no ^•^OH radicals were detected. These mechanistic findings are consistent with recent studies on MOF-perovskite Z-scheme photocatalysts [49,50], where the optimized band alignment ensures efficient charge separation, stronger redox capability and improved pollutant degradation rates. In our case, MIL-53(Fe) contributed a high surface area and abundant active sites for ONP adsorption, while Mn-STO enhanced visible-light absorption and electron mobility. The strong interfacial contact between the two materials facilitates rapid charge transfer across the heterojunction, minimizing recombination and enabling synergistic degradation.

#### 3.4.6. Comparative analysis

Recent studies have highlighted various photocatalysts used for ONP degradation through photocatalysis. To assess the performance of the MIL-53(Fe)/Mn-STO catalyst, it is essential to compare it with previously reported photocatalysts for the removal of nitrophenol. Thus, as shown in table 1, the MIL-53(Fe)/Mn-STO catalyst achieved a remarkable degradation efficiency of 97.75% for ONP, demonstrating superior photocatalytic performance in comparison with other catalysts [51–59] (table 1). This outstanding result can be largely attributed to the direct Z-scheme mechanism of charge transfer, which enhances both the separation of charge carriers and their transfer efficiency.

**Table 1.** Comparison of MIL-53(Fe)/Mn-STO with different photocatalysts reported in existing studies for the photocatalytic degradation of ONP.

composite	concentration (ppm)	light source	time (min)	efficiency (%)	ref.
WO_3_-doped MoO_3_ with ZnO	30	sunlight	180	90.0	[51]
co-doped ZnO	30	UV lamp	180	82.0	[52]
cellulose acetate-doped TiO_2_(CA/TiO_2_)	50	UV light	60	89.0	[53]
Mg_0.1_Zn _0.9_O/L	60	UV irradiation	120	81.0	[54]
sol–gel ZnO	5.8	halogen lamp illumination (300–800 nm)	480	75.0	[55]
Ca^2+^-doped AgInS_2_	15	visible light	120	63.2	[56]
BiVO_4_/Co	150	UV light	75	94.6	[57]
NiFe_2_O_4_ and CuFe_2_O_4_	10	visible light	120	82.0	[58]
ZnO	20	UV lamp	60	95.0	[59]
MIL-53(Fe)/Mn-doped SrTiO_3_	50	xenon lamp	90	97.7	this work

## 4. Conclusion

A novel photocatalyst, MIL-53(Fe)/Mn-STO, featuring a direct Z-scheme heterojunction, was successfully synthesized via a solvothermal approach. This composite exhibited exceptional photocatalytic activity, achieving 97.75% degradation of ONP within 90 min, far surpassing the efficiencies of pristine MIL-53(Fe) and Mn-STO. Scavenger experiments confirmed that h^+^ and ^•^O_2_^−^ radicals were the dominant reactive species responsible for ONP degradation. The high efficiency of MIL-53(Fe)/Mn-STO was attributed to the direct Z-scheme charge transfer mechanism, which enhances both charge separation and migration. Moreover, the composite demonstrated excellent stability and reusability, maintaining its performance over five consecutive cycles. These results highlight MIL-53(Fe)/Mn-STO as a promising and sustainable photocatalyst for water treatment applications. Future work may focus on optimizing the synthesis parameters, testing its performance with other pollutants and scaling up to pilot or real-world wastewater treatment systems to address environmental pollution more effectively.

## Data Availability

The datasets supporting this article have been deposited in Figshare [60]. Supplementary material is available online [61].
